# Antiretroviral Treatment Cohort Analysis Using Time-Updated CD4 Counts: Assessment of Bias with Different Analytic Methods

**DOI:** 10.1371/journal.pone.0027763

**Published:** 2011-11-17

**Authors:** Katharina Kranzer, James J. Lewis, Richard G. White, Judith R. Glynn, Stephen D. Lawn, Keren Middelkoop, Linda-Gail Bekker, Robin Wood

**Affiliations:** 1 Department of Clinical Research, Faculty of Infectious and Tropical Diseases, London School of Hygiene and Tropical Medicine, London, United Kingdom; 2 The Desmond Tutu HIV Centre, Institute for Infectious Disease and Molecular Medicine, Faculty of Health Science, University of Cape Town, Cape Town, South Africa; 3 Department of Infectious Disease Epidemiology, Faculty of Epidemiology and Population Health, London School of Hygiene and Tropical Medicine, London, United Kingdom; 4 Aurum Institute for Health Research, Johannesburg, South Africa; 5 Department of Medicine, Faculty of Health Science, University of Cape Town, Cape Town, South Africa; Boston University, United States of America

## Abstract

**Background:**

Survival analysis using time-updated CD4+ counts during antiretroviral therapy is frequently employed to determine risk of clinical events. The time-point when the CD4+ count is assumed to change potentially biases effect estimates but methods used to estimate this are infrequently reported.

**Methods:**

This study examined the effect of three different estimation methods: assuming i) a constant CD4+ count from date of measurement until the date of next measurement, ii) a constant CD4+ count from the midpoint of the preceding interval until the midpoint of the subsequent interval and iii) a linear interpolation between consecutive CD4+ measurements to provide additional midpoint measurements. Person-time, tuberculosis rates and hazard ratios by CD4+ stratum were compared using all available CD4+ counts (measurement frequency 1–3 months) and 6 monthly measurements from a clinical cohort. Simulated data were used to compare the extent of bias introduced by these methods.

**Results:**

The midpoint method gave the closest fit to person-time spent with low CD4+ counts and for hazard ratios for outcomes both in the clinical dataset and the simulated data.

**Conclusion:**

The midpoint method presents a simple option to reduce bias in time-updated CD4+ analysis, particularly at low CD4 cell counts and rapidly increasing counts after ART initiation.

## Introduction

Observational prospective cohort data of patients on antiretroviral therapy (ART) are often used to estimate the relationship between time-varying CD4+ counts and incident clinical events such as tuberculosis (TB), death, opportunistic infections and malignancies. These studies aim to investigate the effect of actual CD4+ count on morbidity and mortality by using time-varying measures. While within-subject CD4+ count variability [1], [2], [3] will inevitably introduce measurement error, measurement frequency and the choice of when to split time attributed to a certain CD4+ count value might also introduce bias. Measurement frequencies are either determined by the study protocol which specifies time intervals at which individuals are followed (interval cohort) or by prevailing guidelines within the health care service (clinical cohort) [4]. In the latter the frequency of visits and laboratory measurements may also be influenced by the severity of illness, access to and utilization of health care which might increase the bias.

Differences in measurement frequency between two exposure groups have been shown to introduce bias when time to a specific biomarker level is used as a surrogate outcome [5]. The time-point when the CD4+ count is assumed to change might bias effect estimates especially when measurement intervals are wide or CD4+ counts are rapidly changing. A literature review of studies published between January 2006 and August 2010 investigating the effect of time-updated CD4+ counts on mortality and morbidity found that the majority (11/21) of studies did not did not specify the method used to estimate the time-point of change (table 1). Of remaining studies, eight assumed that the CD4+ count remains constant until the date of the next measurement and two studies used linear interpolation between two consecutive CD4+ count measurements to provide a midpoint measurement and time-point.

**Table 1:** Studies conducting analysis using time-updated CD4+.

Author	Journal	Outcome	CD4+ count	Description of how time-updated CD4+ counts were determined.
Dunn[26]	JID	AIDS or death	exposure	Follow-up time from the time that each measurement was obtained was censored at the date of the next measurement.
Guiguet[27]	Open AIDS J	AIDS or death	exposure	CD4+ counts were modeled using linear interpolation between two measurements.
Lawn[25]	AIDS	Death	exposure	Person time was divided into intervals each of which was defined by the CD4+ count measurement at the start of the interval.
Lawn[11]	AIDS	Tuberculosis	exposure	Person-time was subdivided into 4-month intervals for analysis. Each interval was defined by theCD4 cell count measurement at the start of the interval.
Reekie[13]	Cancer	non-AIDS-defining malignancies	exposure	
d'Arminio Monforte[28]	AIDS	death from malignancies	exposure	Each person's follow-up was divided into a series of consecutive 1-months periods, and the individual's status (most recent CD4+ count) was determined.
Lodi[14]	J Natl Cancer Inst	Kaposi sarcoma	exposure	
Engels[29]	JAIDS	Non-Hodgkin Lymphoma	exposure	We considered the most recent laboratory result “current” until the next measurement.
Crum-Cianflone[16]	Arch Intern Med	Cutaneous malignancy	exposure	
Guiguet[30]	Lancet Oncology	Malignancies	exposure	Follow-up was divided into consecutive 1-month periods, and time-varying covariables were updated at the beginning of every month. The CD4+ count was linearly interpolated unless ART was started between 2 measurements.
Podlekareva[31]	Sand J Infec Dis	Fungal infections	exposure	
Prosperi[17]	CID	Malignancies	exposure	
Seyler[18]	AIDS Res Human Retroviruses	Severe morbidity	exposure	
Sogaard[32]	PLoS one	Death from pneumonia	confounder	CD4+ counts were estimated between measurements by carrying forward the value from the most recent measurement
Walker[19]	Lancet	Effect of Co-trimoxazole	confounder	
Crum-Cianflone[15]	AIDS	Malignancies	exposure	
Phillips[33]	AIDS	Death	exposure	Person time was counted from the time of each qualifying CD4+ count until the next CD4+ count.
Beaudrap[20]	BMC Infect Dis	AIDS defining illness	exposure	
Mocroft[21]	AIDS	Clinical disease progression	exposure	
Bohlius[22]	Antivir Ther	Non-Hodgkin Lymphoma	exposure	
Bruyand[34]	CID	Malignancies	exposure	We assumed that the value of the measurement reported at a given follow-up visit remained stable until the next follow-up visit

We aimed to assess how different methods of dealing with time points influence effect estimates and rates using data from a clinical ART cohort with frequent measurements. We used the two methods most frequently used in the literature and investigated the effect of a third method assuming that the CD4+ count remains constant from the midpoint of the preceding interval until the midpoint of the subsequent interval.

The clinical ART cohort used for this study was based in Cape Town, South Africa and CD4+ counts were measured monthly for the first 3 months and 3 monthly thereafter. We also investigated the direction of bias using a simulated dataset.

## Methods

### Data collection

Data collected in a peri-urban township in the greater area of Cape Town as part of the CIPRA-SA trial were used for this analysis [6]. The trial randomized patients to nurse or doctor-monitored HIV care and showed equivalence of the two monitoring strategies for treatment failure over 2 years. A total of 363 HIV-positive ART-naïve patients with a CD4 cell count of ≤350 cell/uL or WHO stage 4 disease from this study community were enrolled in the trial in Cape Town. All patients received a standard ART regimen and were managed according to the South African National Guidelines [7].

CD4+ counts were measured at weeks −4, 0, 4, 8, 12 (relative to the start of ART) and then every 12 weeks. Incident TB was used as the outcome of interest. Start and end of TB treatment were determined by merging the ART register with the electronic TB register on first name, surname, medical record number, date of birth, truncation of names and switching of first name and surname. This method was validated by clinical folder review in a similar dataset of 585 patients from a different study and revealed 96.1% sensitivity and 97.4% specificity. All identifiers were removed from the data after merging.

Individuals who did not live in the study community and individuals who were on TB treatment at ART initiation and died or were lost to follow-up before they completed treatment were excluded from the analysis.

The exposure was time-updated CD4+count and the outcome was incident TB defined as starting TB treatment. Person-time accrued from ART initiation to the date of TB disease, death, becoming lost to follow-up or the 31^st^ December 2008 was calculated. Individuals who were on TB treatment at time of ART initiation were only included in the analysis after they had completed TB treatment. Individuals who developed incident TB were re-included in the analysis after completing TB treatment. Individuals only contributed time while they were on ART and person-time while defaulting care was excluded from the analysis, as neither the exposure (CD4+ count) not the outcome (TB) was known for these periods.

### Time-updated CD4 count

**Figure 1:**
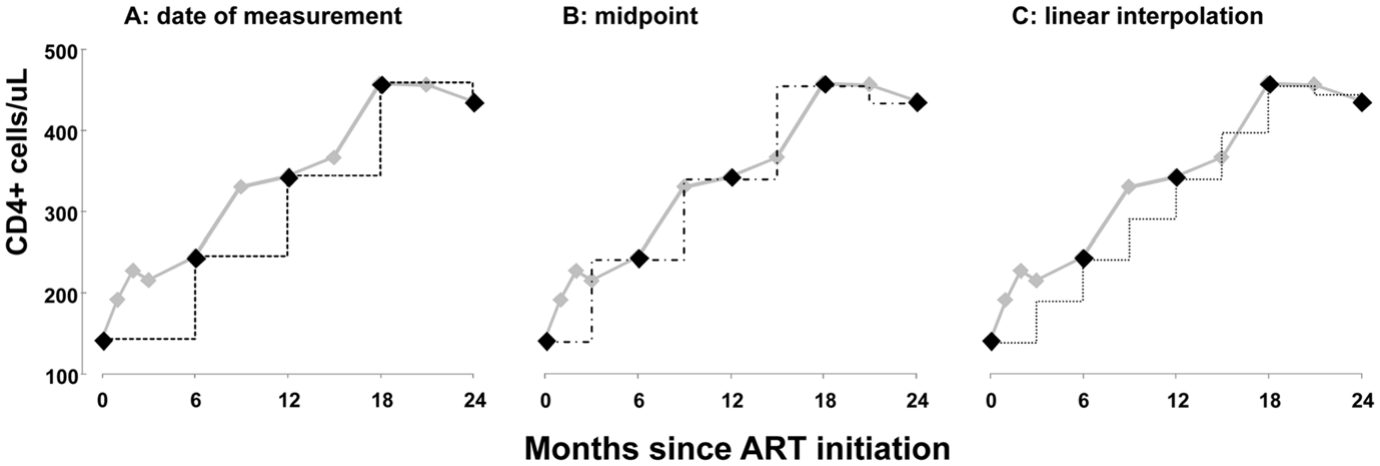
Illustration of the three different methods of modeling CD4+ count. In the patient shown, we actually observed 11 CD4+ cell counts over the two years (grey line). We have illustrated what would have been modeled if only the results at 6 month intervals (black diamonds) had been available. Dotted and dashed lines (black) are the CD4+ counts assumed by the three different methods: data of measurement (A), midpoint (B) and linear interpolation (C).

The data were analyzed in three different ways: the first analysis assumed that the CD4+ count changed at the date when the blood sample for CD4+ count measurement was drawn (date of measurement analysis) (figure 1A); the second assumed a change of CD4+count at the midpoint between two measurements (midpoint analysis) (figure 1B); and the third calculated an additional CD4+ count using a linear interpolation between two consecutive CD4+ measurements and used the date when the blood samples were drawn and the midpoint between the two dates as the time point of change of CD4+ count (linear interpolation analysis) (figure 1C).

### Newly generated datasets

A dataset including baseline CD4+ counts and 6-monthly CD4+ counts only was generated. From this dataset 15% of the follow-up CD4+ counts were randomly selected and removed to simulate the reality of missing data in clinical cohorts. A total of 100 datasets with 15% randomly missing follow-up CD4+ counts were generated.

### Gold standard dataset

The effect estimates and person-time using the newly generated dataset with 6-monthly CD4+ counts and different methods to estimate time-point of change were compared with results obtained when analysing the dataset using all available CD4+ counts (gold standard dataset). The gold standard dataset included CD4+ counts measured on a monthly bases from 0–3 months on ART, followed by 3-monthly CD4+ counts until death, loss-to-follow up, transfer out or censoring. Table 2 shows the difference in median CD4+ counts over time in the gold standard dataset and the 6-monthly dataset using different methods to estimate the time-point of CD4+ change.

**Table 2:** Median CD4 counts and interquartile ranges at baseline, 1, 2, 3, 6, 9 and 12 months follow-up using different methods to estimate the time-point of change of CD4+ count.

Months	All available CD4+ counts	6 monthly CD4+ counts only		
		Date of measurement	Midpoint	Linear interpolation
0	191.5 (109–256)	191.5 (109–256)	191.5 (109–256)	191.5 (109–256)
1	265 (185.5–365.5)	191.5 (109–256)	191.5 (109–256)	191.5 (109–256)
2	296 (198–381)	191.5 (109–256)	191.5 (109–256)	191.5 (109–256)
3	291.5 (198–380)	191.5 (109–256)	307 (213.5–432.5)	248 (158–324)
6	307 (213.5–432.5)	307 (213.5–432.5)	307 (213.5–432.5)	307 (213.5–432.5)
9	313.5 (213.5–432.5)	307 (213.5–432.5)	333 (236–4447)	318 (222–404)
12	333 (236–4447)	333 (236–4447)	333 (236–4447)	333 (236–4447)

### Statistical analysis

All analyses were carried out using Stata version 11.0 (Stata Corp. LP, College Station, TX, United States of America). The association between time-updated CD4+ count and TB was explored describing the rate of incident TB and using crude Kaplan-Meier curves. Cox proportional hazard regression was used to model the relationship between time-updated CD4+ count and TB. Hazard proportionality was assessed by analysis of scaled Schoenfeld residuals.

Events, person-time, rates, hazard ratios and standard errors were determined for the 100 datasets with 15% randomly missing follow-up data. The overall estimates were calculated according to the combination rules described by Rubin [8].

### Simulated CD4+ dataset

Simulated CD4+ count data by time since treatment initiation and baseline CD4+ strata, 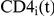, were generated by fitting 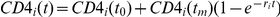 to empirical data from Nash et al.[9] by least-squares, where 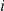 was the CD4+ stratum at treatment initiation, 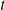
was time since treatment initiation, 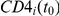 was the average CD4+ level in strata 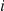 at treatment initiation, 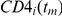 was a parameter determining the increase in CD4+ count in strata 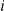 after 5 years of treatment, and 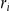 was a parameter determining the rate of CD4+ count increase in strata 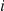. Each CD4+ stratum 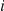 was simulated separately and the results were also combined to generate a ‘mixed’ cohort of 25%, 17%, 18%, 15%, 25% of patients with baseline CD4+ of 25–50 cells/uL, 51–100 cells/uL, 101–150 cells/uL, 151–200 cells/uL and 201–300 cells/uL respectively, to represent a mix of patients seen in a typical clinic. A clinical South African ART cohort was used to determine the proportions of patients in different CD4+ count strata for the mixed cohort [10].

The areas under the CD4+ curve (AUC) were calculated using date of measurement, midpoint or linear interpolation methods with either 6 monthly or 3 monthly measurements. The AUC measures CD4 exposure. It is derived from the actual CD4+ values and the time spent with these values. Rates were calculated assuming constant rates within CD4+ count strata using TB rate estimates from published literature [11], [12].

### Ethical approval

All patients in the CIPRA-SA trial signed informed consent forms. The trial was approved by the University of Cape Town Ethics Committee and Partners Human Subjects Institutional Review Board. The London School of Hygiene and Tropical Medicine Ethics Committee and the University of Cape Town Ethics Committee and Partners Human Subjects Institutional Review Board gave approval for the analysis of the anonymised data.

## Results

### TB incidence and hazard ratios by time-updated CD4+ count using clinical cohort data

Overall TB incidence was 4.9/100 person–years (PY) (95% confidence interval (CI) 3.6–6.8). TB incidence rates were 14.7 in the lowest CD4+ count stratum (≤200 cells/uL), 3.1 in the middle CD4+ count stratum (201–350 cells/uL) and 2.9 in the highest CD4+ count stratum (>350 cells/uL) when using all available CD4+ counts and performing a date of measurement analysis (table 3). The midpoint analysis revealed TB incidence rates of 16.0, 3.1 and 2.8 for the three different CD4+ count categories. The total person-time spent at low CD4+ counts was less in the midpoint analysis compared to the date of measurement analysis.

**Table 3:** Person-time, rates of tuberculosis and hazard ratios for tuberculosis using clinical cohort data and different methods to estimate the time-point of change of CD4+ count.

CD4+ strata (cells/uL)	Date of measurement analysis				Midpoint analysis				Linear interpolation analysis			
**Survival and Cox regression analysis using all available CD4+ counts**												
	Events	PY	Rate	HR	Events	PY	Rate	HR	Not performed*			
≤200	19	128.9	14.7	1	19	119.0	16.0	1				
201–350	8	261.2	3.1	0.26 (0.11–0.61)	8	255.4	3.1	0.25 (0.11–0.55)				
>350	11	378.7	2.9	0.34 (0.15–0.75)	11	394.5	2.8	0.29 (0.13–0.65)				
**Survival and Cox regression analysis using 6 monthly CD4+ counts only**												
	Events	PY	Rate	HR	Events	PY	Rate	HR	Events	PY	Rate	HR
≤200	22	176.6	12.5	1	18	140.2	12.8	1	20	152.1	13.2	1
201–350	10	256.4	3.9	0.41 (0.19–0.88)	13	246.2	5.3	0.52 (0.25–1.08)	12	261.9	4.6	0.45 (0.22–0.95)
>350	6	335.7	1.8	0.25 (0.06–0.66)	7	382.3	1.8	0.24 (0.09–0.62)	6	354.7	1.7	0.22 (0.08–0.60)
**Survival and Cox regression analysis using 6 monthly CD4+ counts and 15% randomly missing**												
	Events	PY	Rate	HR	Events	PY	Rate	HR	Events	PY	Rate	HR
≤200	16.1	184.7	8.7	1	18.7	145.9	12.8	1	16.1	158.6	10.2	1
201–350	14.5	255.8	5.7	0.86 (0.42–1.77)	12.7	245.9	5.2	0.51 (0.24–1.05)	14.5	262.9	5.5	0.73 (0.35–1.510
>350	7.4	326.2	2.3	0.49 (0.19–1.25)	6.6	374.5	1.8	0.23 (0.09–0.59)	7.4	344.8	2.1	0.40 (0.15–1.02)

*Linear interpolation analysis was not performed for the analysis using all available CD4+ counts, as the result was not expected to differ greatly compared to the date of measurement and midpoint analysis.

TB incidence rates and hazard ratios (HRs) were different when using a dataset with 6 monthly CD4+ counts as compared to analysis using all available CD4+ counts (table 3). With all three estimation methods, compared to the results with more frequent measures, rates were underestimated at low and high CD4+ counts, and overestimated at moderate CD4+ counts, with most marked overestimation in the midpoint analysis.

Analyses using a dataset with 6 monthly CD4+ counts and 15% randomly missing follow-up CD4+ counts revealed more extreme variations in rates, but with the same pattern of underestimation at low and high counts, and overestimation at moderate counts (table 3). The differences in rates and HRs compared to the analysis using all available data were most pronounced using the date of measurement analysis, and least pronounced using the midpoint analysis.

### Area under the CD4+ curve using simulated data

The midpoint analysis estimated the AUC most accurately for cohorts with low (25–50 cell/uL), high (151–200 cells/uL) and mixed baseline CD4+ counts (table 4). The date of measurement analysis underestimated the AUC for all cohorts and time-points. The relative difference was most pronounced in cohorts with low baseline CD4 counts and short follow-up (1 year). The date of measurement analysis was less accurate with 3 monthly measurements than the midpoint analysis with 6 monthly measurements.

**Table 4:** Estimated area under the CD4+ count curve using simulated data and different methods to estimate the time-point of change of CD4+ count.

Baseline CD4+ count of the cohort	Time	Cumulative area under the CD4+ count curve				
		True	Date of measurement method 6 monthly CD4+ counts	Date of measurement method 3 monthly CD4+ counts	Linear interpolation method 6 monthly CD4+ counts	Midpoint method 6 monthly CD4 counts
25–50 cells/uL	1 year	145	99	123	120	142
	5 years	1348	1272	1311	1307	1342
51–100 cells/uL	1 year	180	138	160	157	177
	5 years	1435	1368	1403	1399	1430
101–150 cells/uL	1 year	228	186	208	205	225
	5 years	1662	1597	1631	1627	1657
151–200 cells/uL	1 year	282	238	261	258	278
	5 years	1862	1801	1833	1829	1856
201–300 cells/uL	1 year	345	305	326	323	342
	5 years	2180	2121	2152	2148	2274
Mixed	1 year	237	194	216	213	233
	5 years	1704	1639	1673	1669	1699

### TB rates using simulated data

Both the date of measurement and midpoint analysis underestimated TB rates for low CD4+ count strata (<200 cell/uL). Rates were less accurately estimated using the date of measurement analysis compared to the midpoint analysis (table 5). Rates for some CD4 count+ strata could not be determined as no time was spent in those strata. For example a cohort with a baseline CD4 count of 151–200 did not accumulate any person-time in the CD4+ count strata ≤50 and 51–100. In addition cohorts with baseline CD4+ counts of 25–50 and 51–100 did not improve their CD4+ count beyond 400 over the 5 year period and thus did not accumulate any time in higher CD4+ count strata.

**Table 5:** Estimated rates of tuberculosis using simulated data and different methods to estimate the time-point of change of CD4+ count.

CD4+ strata	True rates	Cohort with baseline CD4+ count 25–50 cells/uL		Cohort with baseline CD4+ count 51–100 cells/uL		Cohort with baseline CD4+ count 151–200 cells/uL		Mixed cohort	
		Date of measurement method	Midpoint method	Date of measurement method	Midpoint method	Date of measurement method	Midpoint method	Date of measurement method	Midpoint method
≤50	21.7	11.25	13.2	--	--	--	--	--	--
51–100	12.8	--	--	9.69	10.12	--	--	--	--
101–200	9.27	6.65	9.27	6.24	8.13	5.93	6.39	7.38	9.27
201–300	5.48	5.42	5.73	5.39	5.48	4.75	5.18	5.48	5.48
301–400	4.61	4.61	4.61	4.61	4.65	4.51	4.59	4.61	4.66
401–500	4.23	--	--	--	--	4.23	4.23	--	--

## Discussion

This study shows that the time-point when a CD4+ count is assumed to change influences incidence rates of clinical events during ART and effect estimates in time-updated CD4+ count analysis. The analysis using modeled CD4+ count data showed that the midpoint method gives a better approximation of person-time spent at low CD4+ counts compared to the date of measurement method. The choice of time-point when a CD4+ count is assumed to change had the greatest impact in cohorts with low baseline CD4+ counts and during the first year after ART initiation. While the absolute difference in effect estimates was small when analyzing data with frequent measurements, the choice of time-point was important in data with less frequent and missing measurements. Thus the frequency of measurement and the method used to determine the time-point of change in CD4+ count need to be taken into account when comparing effect estimates from different studies. However, most studies performing survival or Cox regression analysis with time-updated CD4+ count as exposure or confounder variable fail to describe how the time-point of change in CD4+ count was determined [13], [14], [15], [16], [17], [18], [19], [20], [21], [22].

The rate of change in CD4+ count is highest in the first months after initiation of ART [9]. The dataset including all CD4+ counts had a particularly high frequency of measurements in the first three months on ART, with testing done at 0, 4, 8 and 12 weeks. Person-time spent with low CD4+ counts was overestimated in all analyses conducted on a dataset with only 6 monthly CD4+ counts compared to analysis using a dataset with all available CD4+ counts. As a result TB incidence rates were underestimated in the low CD4+ count strata. The difference in person-time spent with low CD4+ counts was smallest in the midpoint analysis, but rates and hazard ratios were nevertheless strongly biased using the dataset with 6-monthly CD4+ counts. The bias was due to a smaller number of events estimated to occur in the low CD4+ count strata, which was probably due to chance and small sample size. The analysis using modeled CD4+ count data showed that the midpoint analysis estimated person-time and rates most accurately. The linear interpolation method estimated person-time and rates more accurately compared to the date of measurement, but less so when compared to the midpoint methods. However, more than one interpolation or possibly daily interpolation is likely to improve the accuracy of these estimates.

Our study confirms and extends the findings of a study from Côte d’Ivoire [23]. In this study by Deuffic-Bruban et al., person-time spent at low CD4+ counts (<50 cells/uL) was highest in the date of measurement analysis and lowest in the analysis assuming that the CD4+ count changed immediately to the level of the next measurement [23]. Estimates of rates of opportunistic infections were highest (249/100 PYs) in the analysis assuming an immediate change, followed by the linear interpolation (210/100 PYs) and date of measurement analysis (130/100 PYs). However this study is not comparable to our study or to routine programmatic data because of the very high frequency of CD4+ counts (median time between the last CD4+ measurements 1–1.8 months) throughout the study (compared to a median of 3 months in our study) which means that the differences between methods will be less pronounced. Deufic-Burban et al. did not compare the results from the original dataset and datasets with less frequent measurements and thus they were unable to assess the extent of bias that would be seen in those situations. In contrast, we used the dataset with frequent measurements as a gold standard and compared it to a generated dataset with only 6-monthly measurements (a dataset comparable to most clinical cohort data). Another further important addition in our study was that we used the midpoint method. This method gave a good approximation to the time spent at low CD4+ count strata.

Most analyses investigating the effect of time-updated CD4+ counts on clinical outcomes use CD4+ count categories [14], [19], [21], [24], [25]. Categorizing a continuous variable such as CD4+ count increases the problem of misclassification since small differences can result in a change in category. Bias introduced by categorization needs to be taken into account when analyzing and interpreting the results from time-updated CD4+ count analysis. Limitations of the clinical cohort analysis are the small sample size, the small number of events and the relatively high baseline CD4+ count. The effect estimates calculated in full analysis were imprecise and the extent of bias due to different methods was uncertain from the clinical cohort analysis alone. However the analysis using modeled data confirmed that person-time at higher CD4+ counts and rates were more profoundly underestimated using the date of measurement method compared to the midpoint method. Missing data was randomly missing in the newly created datasets. This is unlikely to be the case in clinical cohorts, where missingness could be more likely in healthy patients with fewer scheduled visits or sicker patients due to difficulties in accessing care. TB rates within CD4+ count strata were assumed constant in the modeled dataset which might not accurately reflect the reality. Thus estimated TB rates might be even more biased if true TB rates differ according to CD4+ count within CD4+ count strata.

Analysis using time-updated CD4+ counts as exposure or confounder should consider using the midpoint method as a simple way to reduce bias. In addition authors should be encouraged to clearly describe the assumption underlying the time-point of change in CD4+ count and researchers conducting meta-analyses should contact authors to determine the method used.
